# Emerging Role of mTOR Signaling-Related miRNAs in Cardiovascular Diseases

**DOI:** 10.1155/2018/6141902

**Published:** 2018-08-23

**Authors:** Arun Samidurai, Rakesh C. Kukreja, Anindita Das

**Affiliations:** Pauley Heart Center, Department of Internal Medicine, Division of Cardiology, Virginia Commonwealth University, Richmond, VA 23298, USA

## Abstract

Mechanistic/mammalian target of rapamycin (mTOR), an atypical serine/threonine kinase of the phosphoinositide 3-kinase- (PI3K-) related kinase family, elicits a vital role in diverse cellular processes, including cellular growth, proliferation, survival, protein synthesis, autophagy, and metabolism. In the cardiovascular system, the mTOR signaling pathway integrates both intracellular and extracellular signals and serves as a central regulator of both physiological and pathological processes. MicroRNAs (miRs), a class of short noncoding RNA, are an emerging intricate posttranscriptional modulator of critical gene expression for the development and maintenance of homeostasis across a wide array of tissues, including the cardiovascular system. Over the last decade, numerous studies have revealed an interplay between miRNAs and the mTOR signaling circuit in the different cardiovascular pathophysiology, like myocardial infarction, hypertrophy, fibrosis, heart failure, arrhythmia, inflammation, and atherosclerosis. In this review, we provide a comprehensive state of the current knowledge regarding the mechanisms of interactions between the mTOR signaling pathway and miRs. We have also highlighted the latest advances on mTOR-targeted therapy in clinical trials and the new perspective therapeutic strategies with mTOR-targeting miRs in cardiovascular diseases.

## 1. Introduction

Cardiovascular disease (CVD) is one of the leading cause of mortality and morbidity in the world and is a global pandemic threat to human health [1, 2]. Coronary artery diseases (CAD) such as ischemia reperfusion injury (I/R) and acute myocardial infarction (AMI) are the primary forms of CVD that account for the majority of the deaths. Apart from this, additional comorbid factors like diabetes [3, 4], obesity [5], inflammation [6], and atherosclerosis [7] escalate the complication associated with heart disease and increase the incidence of death. The underlying mechanisms involved in cardiovascular complication are complex and multifactorial. The existence of several metabolic perturbations in diseases like diabetes and inflammation further pose a tough challenge in understanding the mechanism and pathology of CVD. These obstacles largely impede our goal to develop an effective treatment against progression of CVD and its prevention. However, current therapies for heart diseases have been substantially improved using integrated genome-based evidences and molecular clues. Our recent understanding of genomics and the regulation of gene expression by noncoding RNAs (ncRNA) during both normal and pathological conditions encourage us in exploring the novel therapies for heart disease with a unique perspective.

In the cardiovascular system, the mechanistic target of rapamycin (mTOR) pathway regulates both physiological and pathological processes in the heart [8]. mTOR is an evolutionarily conserved signaling pathway found in various species including yeast [9, 10], Caenorhabditis elegans [11, 12], drosophila [13, 14], and mammals [15–18]. mTOR is a master regulator of cell metabolism and plays a central role in integrating various signaling network [19]. mTOR participates in the fundamental aspect of cell function and is therefore indispensable for cellular life. It governs several key cellular processes such as nutrient sensing [20–22], protein synthesis [23, 24], cell proliferation [14], and apoptosis [25, 26]. mTOR is also actively involved in the epigenetic regulation of gene expression and control process like aging [27] and autophagy [28]. However, aberrant regulation of mTOR is known to play a significant role in various maladies including cancer [29], diabetes [30], aging [31], and cardiovascular diseases [32]. mTOR plays an important role in normal cardiac development [33–36] and during cardiac pathophysiologic condition [37, 38].

Recent studies have demonstrated that mTOR signaling pathway is profoundly influenced by small noncoding RNAs, and an interplay between these two molecules define a synergistic regulation of gene expression [39–41]. The unique combination and cross talk between mTOR and miRs have opened up research interest from a distinct perspective and to revisit mTOR signaling in the light of miR. The intention of this review article is to highlight our recent understanding on mTOR pathway in cardiovascular system and its coordinated interaction with miRs to fine-tune the regulation of gene expression under both normal and pathological conditions.

### 1.1. Structure, Mechanism, and Function of mTOR Complexes

**Figure 1:**
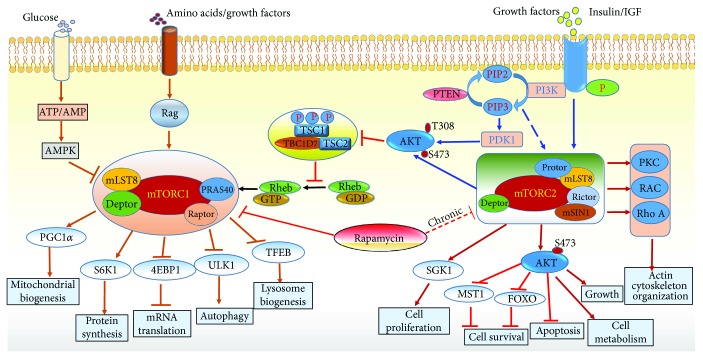
Schematic representation of various subunits of mTORC1 and mTORC2 complex and its upstream signaling regulators and cellular function. The mammalian target of rapamycin (mTOR); insulin growth factor (IGF); adenosine monophosphate activated protein kinase (AMPK); eukaryotic translation initiation factor 4E- (eIF4E-) binding protein 1 (4EBP1); proline-rich AKT substrate 40 (PRAS40); tuberous sclerosis protein ½ (TSC1/2); Ras homolog enriched in brain (Rheb); phosphoinositide 3 kinase (PI3K); Unc-51 like autophagy activating kinase (ULK); ribosomal protein S6 kinase beta-1 (*S6K1*); forkhead box O transcription factor (FOXO); serum/glucocorticoid-regulated kinase 1 (SGK1); peroxisome proliferator-activated receptor gamma coactivator 1-alpha (PGC-1*α*).

The mTOR macromolecular complex is a serine/threonine protein kinase of 289 kDa that belongs to phosphatidylinositol-3-kinase (PI3K) family of proteins and governs several cellular processes including protein synthesis and metabolic regulation [42]. Hall and colleagues first identified target of rapamycin 1 (TOR1) and TOR2 in yeast *Saccharomyces cerevisiae* [43, 44], which was subsequently characterized in mammalian cells and hence called mTOR [42, 45, 46]. Discovered in the year 1970 for its antifungal property [47], rapamycin played a bigger role in elucidating the cellular function of mTOR [42, 48]. The mTOR consists of two major distinct complexes termed as mTORC1 and mTORC2 (Figure 1) and have different sensitivity towards its inhibitor rapamycin [49]. mTORC1 and mTORC2 are similarly large, weighing in at ~1.2 and ~1.4 MDa, respectively [50]. The central core catalytic subunit, mTOR, is common to both complexes and characterized by their own unique subunits. TOR proteins contain ∼2500 amino acids and comprise several distinct domains [51], including 32 tandem HEAT (huntingtin, elongation factor 3, protein phosphatase 2A, and Tor1) repeats towards their N-termini, followed by FAT (FRAP, ATM, and TRRAP) domain consist of multiple antiparallel *α*-helical features, termed tetratricopeptide repeats (TPRs) [51].

mTORC1 consist of five components (1) mTOR (mammalian target of rapamycin), the central catalytic subunit, (2) raptor (regulatory-associated protein of mTOR) [52, 53], (3) mLST8 or G*β*L (mammalian lethal with Sec13 protein 8) [54, 55]. (4) PRAS40 (proline-rich AKT substrate 40 kDa) [56–58], and (5) Deptor (DEP-domain-containing mTOR-interacting protein) [59, 60]. The cryoelectron microscopy structure of human mTORC1 revealed that mTORC1 maintains an obligate dimer with an overall rhomboid shape and a central cavity by interlocking mTOR-raptor interactions [61]. The principal interaction between raptor and mTOR consists of an *α*-solenoid stack formed between the horn and bridge domains of mTOR and the raptor armadillo domain [62].

The precise function of all mTOR-interacting proteins in mTORC1 complex remains to be understood. It was shown that rapamycin forms complex with FKBP12 and interacts with mTOR subunit and inhibits mTORC1 activity [53, 63, 64]. Experimental evidences also suggest that raptor might affect mTORC1 activity by regulating assembly of the complex and by recruiting substrates for mTOR [11, 21, 60]. The role of mLST8 in mTORC1 function is partially understood. Studies using raptor-, rictor-, or mLST8-deficient mice demonstrate that mLST8 is required for mTORC1 activity and is necessary to maintain the rictor-mTOR, but not the raptor-mTOR, interaction [55]. The interaction among different proteins in mTORC1 complexes is crucial and determines its active state. For example, the subunits of mTORC1, PRAS40 and Deptor, are known to interact with each other and docked to the core complex, resulting in the inhibition of mTORC1. PRAS40 and Deptor are phosphorylated by mTORC1, which blocks their interaction leading to the activation of mTORC1 [56–59].

Relatively, much less is known about the regulation of mTORC2 compared to mTORC1. Essentially, mTORC2 contains six different proteins, many of which are components of mTORC1: (1) mTOR; (2) rictor (rapamycin-insensitive companion of mTOR), unique to mTORC2; (3) mammalian stress-activated protein kinase-interacting protein (mSIN1) [65]; (4) protein observed with Rictor-1 (Protor-1); (5) mLST8; and (6) Deptor, an important interacting protein in mTORC1. mTORC2 contains its unique subunit rictor, which is insensitive to rapamycin. This is due to the fact that rapamycin when forms a complex with FKBP12 subunit, it does not bind to mTORC2 and losses it ability to block its activity [66–68]. The N-terminal region of rictor is composed of helical repeat clusters, which binds to mTOR as well as makes multiple contacts with mSin1. The FRB domain of mTOR also shows multiple cross-links with mSin1 and C-terminal regions of rictor. Rictor and mSin1 together generate a steric hindrance to inhibit binding of FKBP12-rapamycin to mTOR, revealing the mechanism for rapamycin insensitivity of mTORC2 [50, 69].

Recent evidences suggest that rapamycin inhibits mTORC1 at low concentration, and a prolonged chronic inhibition leads to inactivation of mTORC2 [70, 71]. Moreover, interaction between rictor and mSIN1 is essential for their own stability and to form the mTORC2 complex, since the deletion of SIN1 blocked the phosphorylation of AKT at serine 473 residue leading to the disruption of rictor-mTOR [72]. Deptor is common to both mTORC1 and C2 and acts as an endogenous inhibitor of mTORC2 [59]. mLST8 is also shown to be a crucial element in the mTORC2 complex formation since the ablation of this protein destabilize mTORC2. Interestingly, several of the subunits in mTORC1 and 2 are common to each other but they interact in an exclusive mechanism that characterize the individual complex. Even though they are unique in many aspects, mTORC1 and mTORC2 phosphorylate entirely different substrates and consequently have distinct function [55].

Several external stimuli such as nutrient, insulin, growth factors, leptin, and stress signals regulate mTOR complexes. However, mTORC1 and C2 respond to these factors differently and have exclusive downstream effects. The primary effector pathway of mTORC1 is through activation of ribosomal proteins S6 kinase 1 and 2 (S6K1/2) by phosphorylating their hydrophobic motif (HM), on Thr389 and Thr 388, respectively, which promotes mRNA biogenesis as well as translational initiation and elongation of protein synthesis. Other substrates of mTORC1 includes 4E- (eIF4E-) binding protein1 and (4EBP1), which are also involved in the activation of gene expression and protein translation. mTORC1 complex is also very sensitive to nutrients, particularly amino acids and glucose level. Deprivation of amino acids especially leucine results in rapid dephosphorylation of S6K1 and 4EBP1 and results in the inactivation of mTORC1 [13, 20]. The energy status of the cell is also sensed by mTORC1 through AMP-activated protein kinase (AMPK). AMPK is phosphorylated in response to low cellular energy status indicated by the high AMP/ATP ratio. The activated AMPK in turn inhibits cell growth via TSC2-dependent suppression of mTORC1 activity and blocks the phosphorylation of S6K1 and 4EBP1 mediated by mTORC1 [22, 73, 74]. Apart from its role in protein synthesis, mTORC1 is also involved in catabolic processes such as apoptosis and autophagy. Under starvation, mTORC1 phosphorylates ULK1 (Unc-51-like autophagy activating kinase), thereby preventing its activation by AMPK, an important activator of autophagy [75, 76].

mTORC2 is widely recognized to play an important role in cell proliferation and response to growth factors such as insulin. Unlike mTORC1, which acts through various downstream effectors, mTORC2 mainly acts through insulin/PI3K pathway via phosphorylation of AKT at serine 473, Thr 308, and Thr 450 residues upon stimulation by insulin [77, 78]. Recent evidence also suggest that mTORC2 can phosphorylate AKT at S377/T479 residues in the C-terminal end and can regulate apoptosis [79]. The mTORC2 subunit mSin1 contains a phosphoinositide-binding PH domain that is critical for the insulin-dependent regulation of mTORC2 activity [65, 80, 81]. Insulin binding to its tyrosine kinase receptor activates IRS and recruits activated PI3K. [82] The PI3-PDK pathway phosphorylates AKT in a mTORC2-dependent manner [83]. mTORC2 phosphorylates several protein kinases including PKA, B, C, G, SGK1 (serum/glucocorticoid-induced kinase 1), and Rho1 (GDP-GTP exchange protein-2), resulting in their stabilization and activation [84–90]. Rictor enables mTORC2 to directly phosphorylate AKT at its Ser473 and facilitates Thr308 phosphorylation by PDK1 (phosphoinositide-dependent kinase 1) as part of the insulin-signaling cascade [91].

Interestingly, there is a cross talk between mTORC1 and mTORC2 and they are functionally interconnected. Apparently, mTORC1 inhibits mTORC2 through phosphorylation of rictor and mTORC2 regulates mTORC1 through phosphorylation of AKT, which controls both the activity and abundance of AKT [92]. Rictor subunit of mTORC2 complex can be phosphorylated by S6K1, a downstream effector of mTORC1, and this phosphorylation negatively regulates the mTORC2-dependent phosphorylation of AKT-S473 [93]. In contrast, upon stimulation with growth factors, mTORC2 activates AKT, which in turn enhances mTORC1 activity through the inactivation of TSC1/2 (tuberous sclerosis complex). The TSC2 is inactivated by AKT-dependent phosphorylation, which destabilizes TSC2 and disrupts its interaction with TSC1 [94, 95].

### 1.2. MicroRNA Biogenesis and Mode of Action

MicroRNAs (miRs) are small noncoding RNAs consisting of approximately 22 nucleotide in size and function as gene suppressors [96, 97]. They bind to the 3′ untranslated region (UTR) of mRNA and regulate their expression via either degradation of mRNA transcript or interfere in the translation process [98, 99]. The regulation of gene expression mediated by miR has now been widely recognized as a major molecular mechanism employed by cells to control various function and signaling pathway [100–103], including AKT, AMPK, JNK, and TGF-*β* [104–114].

**Figure 2:**
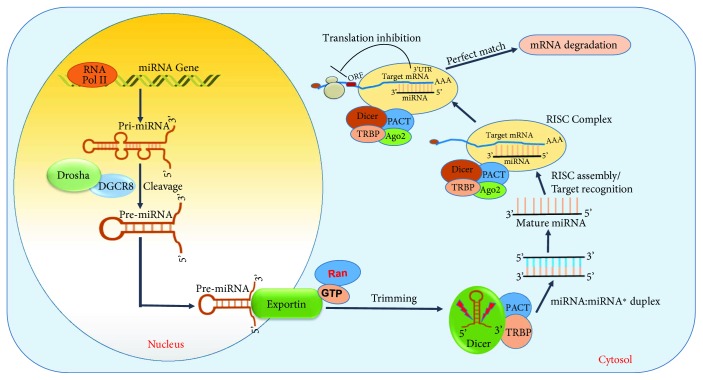
Illustration of miRNA biogenesis pathway and mode of gene regulation. RNA polymerase (RNA-Pol); Drosha; DiGeorge syndrome critical region-8 (DGCR8); guanosine triphosphate (GTP); RAs-related nuclear protein (Ran); human immunodeficiency virus trans-activating response RNA-binding protein (TRBP); argonaute 2 (Ago2); RNA-induced silencing complex (RISC); protein kinase RNA activator (PACT); open reading frame (ORF); 3′ untranslated region (3′UTR).

miRs are encoded across genomic locations including introns and intergenic. Once synthesized and matured through several steps these miRs bind to the complementary 3′UTR of their target mRNA and either degrade or silence them [115–117]. miRs undergo a series of maturation process before they develop into a mature miR **(**Figure 2**)**. They are initially synthesized from their respective genomic region by the enzyme RNA polymerase II into a hairpin structure of approximately ~400–500 bps nucleotide into a primary miR transcript [118, 119]. They are further cleaved by the enzyme Drosha into a 70 nt length nucleotide, named pre-miR [120, 121]. The pre-miR then binds with the protein exportin-5 which transports them out of the nucleus for further trimming [122]. Once in the cytoplasm, they undergo further cleavage by the enzyme ribonuclease III (RNase III) and dicer in to a 22 nt mature miR [123–125]. The mature miR, depending on the complementary nucleotide sequence of its seed region (2–7) at its 5′ end, forms complex with its target mRNA. The double stranded miR-mRNA complex induces the RNA-induced silencing complex (RISC) and targets them for degradation or gene suppression [126–128]. A near perfect match between the seed region (~8 nt) of miR and mRNA leads to a complete degradation of the mRNA, while a partial complementarity results in the suppression of the gene expression [129–133]. miRs are transcribed either as an individual miRNA (e.g., miR-1) or as a family of clusters (e.g., miR-17~92) [134]. The coding region for miRNA can arise from either strand of the DNA and can have multiple mRNA targets [135–137].

## 2. mTOR in Cardiovascular Diseases

**Figure 3:**
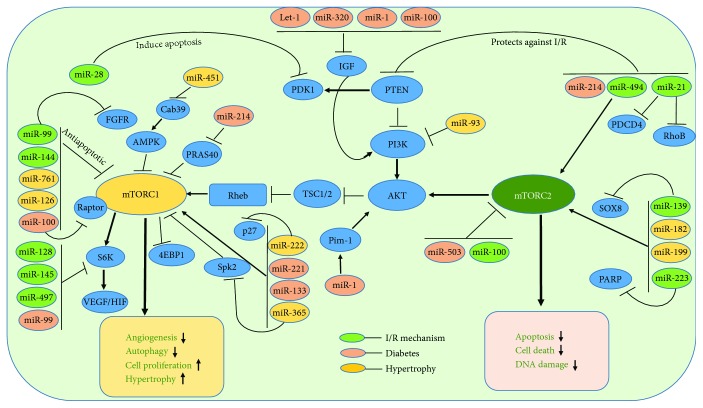
Diagram depicting network of coordinated interaction between miRNA and mTOR pathway in the regulation of cardiovascular diseases. Fibroblast growth factor receptors (FGFR); insulin-like growth factor 1 (IGF-1) sphingosine kinase 2 (Spk2); regulation of proline-rich AKT substrate 40 kDa (PRAS40); phosphatidylinositol-4,5-bisphosphate 3-kinase (PI3K); phosphoinositide-dependent kinase l (*PDK1*); calcium-binding protein 39 (Cab39); poly (ADP-ribose) polymerase (PARP); SRY-related HMG-box 8 (SOX8); programmed cell death protein 4 (PDCD4); Ras homolog gene family, member B (RhoB); phosphatase and tensin homolog (PTEN); vascular endothelial growth factor (VEGF); hypoxia-inducible factors (HIF); tuberous sclerosis protein (TSC1/2); ras homolog enriched in brain (Rheb); ribosomal protein S6 kinase beta-1 (S6K1); proviral integration site for Moloney murine leukemia virus-1 (Pim-1).

The role of mTOR in cancer and aging is well recognized and documented with numerous scientific publications [35, 138–144]. However, their role in cardiovascular system is still in the early stages and yet to be elucidated. mTOR plays an important role in the normal development of cardiovascular system and is crucial during pathophysiological conditions [35, 145–150]. Nevertheless, studies have revealed a novel role for mTOR in CVDs like ischemia reperfusion (I/R) injury [151–153], heart failure [154–156], and its associated risk factors including diabetes [146, 157, 158] and aging [31, 159, 160]. Recent research findings also indicate that the components involved in mTOR pathway are regulated by miRs in cancer and other diseases [39, 40, 161, 162]. A comprehensive role of miRs regulating mTOR signaling in cardiovascular diseases is depicted in Figure 3.

### 2.1. miRNA-Dependent Regulation of mTOR in Ischemia Reperfusion Injury

Oxidative stress induced by ROS generation is a major mechanism of cell injury during myocardial I/R injury [163]. Deprivation of oxygen during I/R stress activates mTORC1 [146, 151, 158, 164] and controls several downstream kinases and leads to cellular effects such as apoptosis [165], autophagy [166, 167], and proliferation [22]. Accumulating evidences point out several different miRs are involved in the modulation of mTOR signaling via targeting mTOR-interacting partners [40, 168–171].

mTOR mediates cardiomyocyte response during ischemia and is an important determinant of cell survival [172, 173]. Inhibition of mTORC1 was shown to be beneficial for the survival of cardiomyocytes via induction of autophagy [174]. Sciarretta et al. demonstrated that selective and direct mTORC1 activation is detrimental during acute cardiac energy deprivation, whereas both pharmacological and genetic mTORC1 inhibition are protective [175]. Pretreatment with rapamycin, the mTOR inhibitor, reduced myocardial infarct size after I/R injury by attenuating necrosis and apoptosis in cardiomyocytes [158, 176]. Reperfusion therapy with rapamycin also attenuated myocardial infarction and apoptosis by activation of PI3K and ERK [177]. Activation of autophagy prevents unwanted expenditure of cellular energy to damaged cells, especially mitochondria, which results in an increased ROS generation [174, 178]. Cardiac-specific overexpression of Rheb leads to the inhibition of Atg7, a key effector protein in the autophagy cascade, and enhances cardiomyocyte cell death through activation of Rheb/mTORC1 signaling pathway [175]. Interestingly, mTOR inhibition with rapamycin promotes the survival of oxygen-deprived cardiomyocytes through activation of autophagy via inhibition of Ras homolog enriched in brain (Rheb) protein [175]. These results indicate that Rheb is a main regulator of mTORC1 during cardiomyocyte energy stress, and Rheb/mTORC1 inhibition promotes cell survival through activation of autophagy [175]. Moreover, obesity and metabolic syndrome, which are characterized by increased myocardial susceptibility to ischemic injury and cardiovascular mortality, are associated with inadvertent activation of the Rheb/mTORC1 pathway and reduction of autophagy [175, 179–181]. Mice with high fat diet- (HFD-) induced obesity and metabolic syndrome exhibit deregulated cardiac activation of Rheb/mTORC1 and inhibition of cardiac autophagy, which lead to increased ischemic injury [175]. Rapamycin treatment before prolong ischemia (3 hours) increases autophagy and significantly reduces the myocardial infarction of both HFD-treated mice [175].

Autophagy is a delicate process that involves a closely-knit transcriptional and epigenetic regulation through miRs [182, 183]. Overexpression of microRNA-99a (miR-99a) through intramyocardial delivery improved cardiac function after MI stress and prevented cell death via enhancing autophagy in mTOR/P70/S6K-dependent signaling pathway [184]. Notably, overexpression of miR-99a in the border zone of infarct area prevented cell apoptosis, but increased autophagy via inhibiting mTOR/P70/S6K [184]. Furthermore, the expression level of miR-99a was reduced in neonatal mice ventricular myocytes (NMVMs) subjected to hypoxia. Similarly, intramyocardial delivery of lenti-miR-99a in mice showed a significant improvement in both left ventricular (LV) function and cell survival post four weeks of MI compared to sham groups [184]. Even though the study illustrated that miR-99a is cardioprotective through decreasing mTOR activity, it did not show a direct target of miR-99a. However, further evidence from the same group also showed that fibroblast growth factor receptor 3 (FGFR3) to be a direct target of miR-99a and hinted a possible role for FGFR-mTOR pathway in MI-induced hypertrophy [185]. Interestingly, multiple findings established a link between miR-99a and FGFR in cell proliferation via mTOR signaling [186–188].

Prevention of the loss of cardiomyocyte during I/R injury is the primary focal point and strategic approach to avoid cardiac dysfunction post-MI, which can potentially be achieved by regulating apoptosis and autophagy [189]. Experiments using primary neonatal cultured mouse cardiomyocytes identified a direct link between miR-28 and PDK1, an immediate upstream regulator of mTOR in the PI3K pathway [190]. Induction of oxidative stress in cardiomyocyte using hydrogen peroxide elevated the expression of miR-28 and increased apoptosis-mediated cell death [190]. Interestingly, overexpression of miR-28 downregulated p-AKT, p-p70, and p-mTOR suggesting a direct interference of mTOR signaling by miR-28 [190]. Mechanistically, PDK1 was found to be a direct target of miR-28 and target-binding assay using luciferase activity and PDK protein expression after transfection with miR-28 confirmed the prediction [190]. Similarly, miR-223 has been reported to play an important role in cell survival by regulation of autophagy and apoptosis [191]. The miR-223 was upregulated in the border zone of infarct area in rats subjected to LAD occlusion [191]. Moreover, overexpression of miR-223 protected H9c2 cells and neonatal rat cardiomyocytes (NRCMs) against hypoxia-induced apoptosis by directly targeting PARP-1 [191]. Decisively, this study showed that H9c2 and NRCMs cells treated with miR-223 mimic increased p-AKT and p-mTOR expression under hypoxic conditions and the protective effect of miR-223 was abolished upon treatment with miR-223 inhibitor [191]. Although, earlier study by van Rooij et al. reported an upregulation of miR-223 in human failing heart tissues [192], but did not explore on the mechanism. The same study showed that miR-29 family are downregulated in the region of the fibrotic scar after MI.

Phosphatase and tensin homology deleted from chromosome 10 (PTEN) are an important determinant for the activation of AKT through PI3 kinase-mTOR pathway. It enhances cardiomyocyte cell death and increases cardiac dysfunction during MI and I/R injury [193–196]. Genetic ablation or pharmacological inhibition of PTEN has been shown to be cardioprotective against MI and vascular remodeling [196, 197]. Our laboratory demonstrated that miR-21 had a powerful cardio protective effect against I/R injury [198–200]. miR-21 expression is induced in the border zone of the infarcted hearts, but it is significantly decreased in the infarcted area [181, 192]. Overexpression of miR-21 protects against I/R injury by reducing myocardial infarct size and apoptosis, by its target genes, PTEN, and programmed cell death 4 (PDCD4) [201–203]. Recent studies suggest that the therapeutic effects seen with miR-21 may be mediated through PTEN/AKT/mTOR signaling pathway [204]. It was observed that miR-21 expression was downregulated, and autophagy was remarkably increased in H9c2 cells during H/R injury. Simultaneously, increased apoptosis after H/R injury was associated with reduction of Bcl2-Bax ratio. Such an effect was abolished by overexpression of miR-21 with a miR-21 precursor, which also inhibited autophagic activity and decreased apoptosis accompanied by the activation of the AKT/mTOR pathway [204]. Thus, it appears that miR-21 plays an active role in disrupting the PTEN-AKT-mTOR pathway. Similar result was also observed in cardiac stem cells, where miR-21 reduced hydrogen peroxide- (H_2_O_2_-) induced apoptosis, as evidenced by the downregulation of caspase-3 and Bax and upregulation of the antiapoptotic Bcl-2 [205]. Overexpression of miR-21 suppressed the expression of PTEN, a direct target of miR-21, with simultaneous increased in the phosphorylation state of AKT. Moreover, the antiapoptotic effect of miR-21 was abolished in cells treated with miR-21 inhibitor and PI3 inhibitor, LY294002, suggesting an involvement of PTEN/PI3K/AKT signaling in miR-21-mediated antiapoptotic effect [205]. Although miR-21 has been considered to promote cellular proliferation, invasion, and migration in various types of tumors [206, 207], rapamycin treatment induced the expression of miR-21 in human umbilical vein endothelial cells (HUVECs) but attenuated endothelial cell proliferation and migration [208]. RhoB, an important partner in AKT-mTOR pathway, is a direct target of miR-21, and silencing of RhoB impairs endothelial cell migration and tubulogenesis, thus providing a possible mechanism for miR-21 to inhibit angiogenesis after rapamycin treatment [208, 209]. However, raptor knockdown, but not rictor silencing, upregulates miR-21 expression, and inhibition of miR-21 blunted the antiproliferative and antimigration effects of rapamycin treatment [208].

Interestingly, miR-21 was shown to be upregulated with rapamycin treatment in angiomyolipoma-derived cells isolated from patient with lymphangioleiomyomatosis (LAM) [210]. Conceptually, LAM is induced by mutation in the tuberous sclerosis complex genes (*TSC1* or *TSC2*) [211] and results in hyperactivation of mTOR signaling, which is characterized by proliferation of smooth muscle-like cells and leads to the malfunction of the lungs. The study demonstrated that 19 miRNAs were differently regulated by rapamycin, and miR-21 was shown to be robustly upregulated in TSC2-deficient 621–101 cells (Renal angiomyolipoma cells). This study also suggested that rapamycin-mediated upregulation of miR-21 is independent of AKT signaling, but rather dependent on mTOR. More importantly, the study also demonstrated that rapamycin potentiates the Drosha-mediated posttranscriptional processing of pri-miR-21 to pre-miR-21. Moreover, rapamycin was clinically shown to improve pulmonary function in LAM patients. Surprisingly, a recent another study demonstrated a significant upregulation of miR-21 in Tsc2-deficient cells compared to wild type controls, which was further induced by rapamycin [212]. Experimental evidences suggest that miR-21 induced proliferation, tumor growth, and offered resistance to apoptosis in TSC2-deficient cells. Moreover, data analysis of RNA Seq implicated that miR-21 promoted mitochondrial adaptation and homeostasis in Tsc-2-deficient cells. Inhibition of miR-21 using LNA reduced the tumor size and mitochondrial function. In addition, rapamycin cotreatment with miR-21 inhibition more efficiently reduced tumorigenic growth of Tsc2-deficient cells *in vivo* xenograft model. Importantly, this study showed that rapamycin increased mitochondrial content and polarization, and these effects of rapamycin were miR-21-dependent. The study also proposed that the unexpected upregulation of miR-21 in TSC2-deficient cells was partly due to miR-21 regulation of mTOR in a noncanonical pathway via either STAT3 or Rheb [212]. Recently, mTORC1 was also reported to regulate the miRNA biogenesis pathway itself [213]. Extensive expression analysis of 752 miRs in TSC2-deficient cells, treated with Torin1 (inhibitor of both mTORC1 and C2), demonstrated an upregulation of majority of miRs in consistent with the increased activity of microprocessor (the multiprotein complex that includes Drosha (a type III RNAse) and DGCR8) in TSC2-deficient cells. Microprocessor activity is regulated in part by GSK3*β*, which is phosphorylated at S9 and subsequently inhibited by mTORC2 via AKT. Inhibition of mTORC1 impaired the microprocessor activity through regulation of Drosha and GSK3*β*-dependent pathways via mTORC2 [213].

Numerous studies reported that miR-21 is involved in a variety of disorders and is highly upregulated during cardiac remodeling [201, 214–216]. However, genetic deletion of miR-21 or acute inhibition of miR-21 did not alter the pathological responses of the heart to pressure overload or other stresses, which suggests that miR-21 is not required for cardiac hypertrophy, fibrosis, or loss of contractile function in response to acute or chronic injury in mice [217]. However, the precise role of miR-21 regulating mTOR signaling in cardiovascular system is still not well evolved, and the effect of miR-21 on proliferative cells like endothelial, smooth muscle cell, and nonproliferative cells like cardiomyocytes may be different. However, several reports in cancer biology strongly suggest an active role for miR-21 in regulating mTOR signaling largely through PTEN/PI3K/AKT pathway [218–222].

Angiogenesis is an important process that restores blood supply to the infarct area post-MI and I/R injury and improves cardiac function [223, 224]. In this context, miR-100 was reported to be an antiangiogenic miR and functioned through repressing mTOR signaling after induction of hind-limb ischemia in mice [162]. miR-100 modulated proliferation, tube formation, and sprouting activity of endothelial cells and migration of vascular smooth muscle cells and functions as an endogenous repressor of mTOR. Inhibition of miR-100 by specific antagomirs stimulated angiogenesis with functional improvement of perfusion after femoral artery occlusion in mice. Moreover, the stimulatory effect of antagomir therapy was abolished by simultaneous rapamycin treatment, demonstrating that the angiogenic effect of miR-100 inhibition in hind-limb ischemia was dependent on its target gene mTOR [162]. Nevertheless, this study did not address the specific role of mTORC1 or C2 in blocking the angiogenic response and did not use a long-term treatment with rapamycin [162].

In tumor glioma, miR-128 is downregulated and acts as a tumor suppressor by directly targeting p70S6K1 [225]. Overexpression of miR-128 attenuated cell proliferation, tumor growth, and angiogenesis by suppressing p70S6K1 and its downstream signaling molecules such as HIF-1 and VEGF expression. Similarly, the expression of miR-145 is downregulated in colon and ovarian cancer, and overexpression of miR-145 inhibits tumor growth and angiogenesis by targeting p70S6K1 and suppressing its downstream angiogenic factors HIF-1 and VEGF [226]. Another miR, miR-497 is downregulated in breast, cervical, head-and-neck, colorectal, and prostate cancers, and overexpression of miR-497 sensitizes the resistant ovarian tumor to cisplatin treatment by targeting mTOR and p70S6K1 [227]. Among the three miRs previously reported to target p70S6k1, only miR-128-3p is downregulated in human cardiomyocytes during H/R by Tongxinluo (TXL, a traditional Chinese medicine, widely used to treat cardiovascular and cerebrovascular diseases). Interestingly, TXL restored p70S6K1 but had no effects on miR-145-5p and miR-497-5p [228]. Inhibition of miR-128-3p activated mTOR via increasing the phosphorylation and abundance of p70s6k1.

PI3K/AKT/mTOR pathway has been shown to be suppressed by miR-139 in I/R injury in H9c2 cell line [229]. The overexpression of SOX8, a target of miR-139, alleviates hypoxia-induced cell injury via activation of PI3K/AKT/mTOR and MAPK pathway [229]. Recently, several miR profiling studies revealed that miR-494 was downregulated in human failing hearts as well as ischemic/hypertrophic hearts of animals [230–232]. The cardiac-specific overexpression of miR-494 in mice protected hearts against I/R-triggered injury; conversely, knockdown of endogenous miR-494 by antagomir sensitized hearts to I/R-induced injury [233]. The overexpression of miR-494 suppressed the levels of proapoptotic proteins (PTEN, ROCK1, and CaMKII*δ*) after I/R injury, which also induced AKT signaling in concert, a critical survival pathway in the myocardium mediated through mTORC2 activation [233]. Also, the inhibition of miR-494 using antagomir elevated the level of PTEN while simultaneously suppressing the level of pAKT (S473) after I/R injury [233].

Apart from the intracellular regulation of mTOR pathway by miRs, miRs packed in exosomes can affect cardiac function. Based on the miRNA array data, remote ischemic preconditioning (rIPC) altered the myocardial expression of miR-144 in mice [234]. Initially, it was shown that rIPC increased the myocardial expression of miR-144, whereas I/R injury alone significantly reduced the level of miR-144. Intriguingly, the exosomes isolated from tissue samples of rIPC hearts were rich with the expression of miR-144 upon rIPC. Moreover, intravenous administration of miR-144 via tail vein injection induced early and delayed cardioprotection in Langendorff isolated perfused model of I/R injury [234]. This study also showed that miR-144 directly targeted mTOR as evident with the decreased p-mTOR and increased autophagy signaling upon miR-144 administration. More precisely p-AKT (S473), a marker for mTORC2 activation, was increased in the heart upon miR-144 injection in mice [234]. These finding suggest that miR-144 acts via suppressing mTORC1 while simultaneously activating mTORC2 complex [234]. In silico analysis of miRNA-target mRNA prediction algorithm (TargetScan 6.0) revealed two specific miR-144 binding sites in the mTOR 3′UTR region with perfect Watson–Crick matches at miRNA positions 1–7 and 2–8 [235]. The interaction of miR-144 and mTOR and its clinical significance have been evaluated in human cancer biology. Specifically, the downregulation of miR-144 leads to poor prognosis of cancer patients via activation of the mTOR signaling pathway [235].

### 2.2. Regulation of mTOR through miRNA in Diabetes and Obesity

Diabetes is a major risk factor for CVD and is characterized by elevated blood glucose, insulin resistance/deficiency, and metabolic abnormalities [236, 237]. Since mTOR is sensitive to nutrient, excessive glucose level in the blood stream activates mTOR [146, 151, 158, 238]. Prolonged activation of mTORC1 induces insulin resistance in adipose tissue through the S6K1-mediated inhibition of insulin signaling that disrupts the recruitment and activation of PI3K via phosphorylation of insulin receptor substrate-1 (IRS-1) [239, 240]. Similar aberrant mechanism in cardiovascular tissues, in conditions like diabetic and obesity, leads to cardiac abnormalities through S6K1-IRS-1 [241] and its effector kinases like MAPK [242], AMPK [241], and glycogen synthase kinase-3*β* (GSK3*β*) [148, 243, 244]. Several miRNAs were identified to play a role in diabetes by regulating insulin signaling and glucose metabolism [238, 245]; [246, 247]. Some of the prominent miRs that regulate mTOR pathway are miR-133a, miR-100, miR-221, miR-483-3p, miR-133a, miR-503, miR-214, microRNA-99a, miR-143, miR-126, and miR-181a-5p.

Inhibition of Let-7 family of miR was shown to be beneficial and promoted cardiac function against I/R injury in diabetic rats [248]. I/R injury in diabetic rat significantly increased let-7 miR as well as infarct size, while antagomir let-7-treated diabetic group offered protection against I/R [248]. Moreover, the myocardial expression of IGF1 and GLUT4 as well as p-AKT (S473) were significantly lower with activation of mTOR in diabetic group. Notably, blocking of let-7 expression or treatment with rapamycin effectively increased AKT phosphorylation at S473 residue, while simultaneously blocked mTOR phosphorylation [248]. IGF plays an important role in glucose metabolism and in the development of insulin resistance, which are crucial events in diabetic cardiomyopathy. miR-1 has been shown to directly targeted IGF-1 [249] and regulated PI3-AKT pathway [250]. In support of this notion, it was shown that miR-1 increased during diabetic cardiomyopathy, which led cardiomyocyte apoptosis through targeting Pim-1 (proviral integration site for Moloney murine leukemia virus-1) [251]. Inhibition of miR-1-dependent downregulation of Pim-1 using miR-1 antagomir resulted in the elevation of phosphorylated AKT and abrogation of diabetic-induced cardiac apoptosis [251]. Similarly, miR-320 is also identified to directly target IGF-1 and VEGF and impairs angiogenesis in myocardial microvascular endothelial cells (MMVEC) isolated from Goto-Kakizaki (GK) diabetic rats [252]. Published studies also demonstrated that miR-99a suppressed the expression of IGF-1 and inactivated mTOR in vascular smooth muscle cells (VSMC) [253]. The hyperinsulin-mediated proliferation and migration of VSMC were reversed by overexpression of miR-99 or inhibition of mTOR. Moreover, overexpression of miR-99a reduced AKT and ERK1/2 activity while suppressing p70S6K, a downstream target of mTORC1 [253].

miR-133a is one of the predominantly expressed miRs in the cardiac tissue, which plays a protective role against pathological remodeling by inhibiting cardiac hypertrophy and cardiac fibrosis in diabetes [254, 255]. Studies in the murine model show that diabetes attenuates miR-133a in hearts [256, 257]. Additionally, a diabetic heart failure (DHF) patient population study showed that the attenuation in the level of miR-133a in diabetic hearts was associated with the exacerbation of autophagy and hypertrophy and suppression of mTOR [258]. In contrast, another interesting study conducted to evaluate the cardiac dysfunction in the offspring of maternal diet-induced obesity revealed a role for miR-133a in cardiac hypertrophy [259]. The results showed that the level of miR-133 is significantly increased in ventricular tissue of the Mat-Ob group and cardiac hypertrophy in the offspring [259]. Most notably, AKT1-Ser473 phosphorylation as well as levels of phospho-ERK1/2, phospho-mTOR, and phospho-p38MAPK were significantly elevated in the Mat-Ob group [259], suggesting an active role of mTOR in the development of cardiac hypertrophy upon diet-induced maternal obesity [259].

Elevated levels of fatty acids and glucose observed in obesity and diabetes mellitus (DM) contribute to systematic inflammation [260, 261]. Blood miRNAs signatures in patients with diabetes with/without obesity revealed a significant reduction of circulating miR-100 in obese normoglycemic subjects and subjects with T2D compared to healthy and lean individuals [262]. Visceral adipose miR-100 was also lower in obese patients with T2D compared to those without. Reduced miR-100 levels were associated with adverse metabolic indices, which may lead to the differentiation of fat tissues and subsequent lipid accumulation, potentially contributing to increased obesity. miR-100 led to the differentiation of adipocytes by modulating its direct target IGFR (insulin growth factor receptor), mTOR, and vLDLR signaling.

A recent study characterized the function of the endothelial-enriched miR-100 during vascular inflammation and atherogenesis [263]. It was reported that miR-100 directly repressed several components of mTORC1-signaling, including mTOR and raptor, which led to the stimulation of endothelial autophagy and diminished activity of the proinflammatory transcription factor NF-*κ*B. In a low-density lipoprotein receptor-deficient atherosclerotic mouse model, inhibition of miR-100 enhanced atherosclerotic plaque formation and a higher macrophage content of the plaque, whereas miR-100 mimic attenuated atherogenesis in the aortic root and in the abdominal aorta. Moreover, miR-100 mimic suppressed mTOR and the transcription factor SREBP-2, which subsequently controlled lipid metabolism in hepatocytes. mTOR inhibition with rapamycin showed anti-inflammatory effects through decreasing the expression of E-Selectin, intracellular adhesion molecule 1 (ICAM-1), and vascular cell adhesion molecule-1 (VCAM-1) in response to endothelial cell activation with TNF-*α*. In addition, rapamycin abolished the effects of miR-100 inhibition with TNF-*α* on endothelial adhesion molecule protein expression, confirming the essential role of intact mTOR signaling in the anti-inflammatory effects of miR-100 [263].

Vascular remodeling and cardiac hypertrophy is one of the adverse effect of diabetes and results in end-stage heart failure [264, 265]. To address this phenomenon, cardiac hypertrophy was induced by angiotensin II (Ang II) treatment in diabetic OVE26 mice, and the role of miR-221 on autophagy was investigated [266]. The results demonstrated that Ang II treatment increased the phosphorylation of c-Jun, JNK, mTOR, and miR-221, while decreasing the level of p27, a direct target of miR-221 and regulator of p-mTOR [266]. Direct downregulation of p27 by miR-221 led to mTOR activation and diminished cardiac autophagy of diabetic OVE26 and/or Ang II-treated mice, resulting in cardiac hypertrophy [266].

mTOR plays a contrasting role in type I DM, where there is an insufficient insulin secretion due to deficient pancreatic *β*-cells. In gestational diabetes mellitus (GDM), it was shown that knockdown of miR-503 enhanced insulin secretion of pancreatic *β*-cells, promoted cell proliferation, and protected cells from apoptosis [267]. mTOR has been identified as a direct target of miR-503, and suppression of miR-503 improves insulin secretion and pancreatic *β*-cells proliferation [267]. The regulation of mTOR pathway by miR is also evident in renal cortex of type 1 diabetic mice [268]. Elevation of miR-214 under high glucose conditions decreased the levels of its target PTEN and increased AKT activity (p-S473) and led to phosphorylation of its substrates glycogen synthase kinase-3*β* and phosphorylation of PRAS40. In contrast, antimiR-214 blocked the phosphorylation of both AKT and PRAS40 and attenuated renal cell hypertrophy, suggesting that inactivation of both mTORC1 and C2 is beneficial [268]. Consistent with this finding, studies using placental tissue from women with GDM demonstrated a robust activation of both mTORC1 and C2 as evident with the increased phosphorylation of AKT (S473), (4EBP1), and p70 S6 kinase (S6K) [269]. Data also showed that miR-143 was significantly high using placental tissue and trophoblast cells, and it impaired mitochondrial respiration via targeting hexokinase (HK), a rate-limiting enzyme in glycolysis [269]. Similarly, miR-99a has been shown to be involved in insulin-dependent glucose consumption in human liver cells (HLL7702) via directly targeting mTOR [270]. Cells treated with insulin suppressed the level of miR-99a while increasing glucose consumption and activation of mTOR. In contrast, the overexpression of miR-99a or rapamycin treatment reversed insulin-mediated glucose utilization [270].

### 2.3. Interaction of mTOR and miRNA in Vascular Remodeling and Hypertrophy

Given the role of mTOR in regulating protein synthesis through S6K [271] and cell cycle control [272, 273], it is well established that mTOR play a key role in cardiac hypertrophy [274–276]. In fact, several reports support this notion as mTOR inhibitors have antihypertrophic property [277–279]. Due to its antiproliferative properties, mTOR inhibitors have also been approved as anticancer drugs [280–282]. Intriguingly, the identification of miRNAs as novel emerging regulators of mTOR signaling has provided new insights into a multitude of biological processes, especially in tissue remodeling and hypertrophy, which has been appreciated by the scientific community in cardiac physiology [103, 169, 185]. Hypertrophic stimuli such as phenylephrine [283], angiotensin II (Ang II) [37, 284, 285], and endothelin-1 [286] are known to activate mTORC1 in the heart and result in robust vascular remodeling leading to heart failure [274]. However, mTORC2 is essential for the preservation of cardiac function and attenuation of pressure overload-induced cardiac hypertrophy [287]. It is increasingly apparent that mTOR [156, 283] and miR [216, 254] have a critical role in the development of cardiac hypertrophy and it is becoming important to understand the mechanism by which these two major regulators communicate with each other.

Cardiomyocyte-specific miR-199a overexpression inhibited autophagy and induced cardiac hypertrophy via targeting glycogen synthase kinase 3*β* (GSK3*β*) involving mTOR signaling [39]. The mTOR signaling was activated in miR-199a transgenic hearts [39]. In addition, treatment with rapamycin blocked the activation of p-mTOR and p-S6 in miR-199 transgenic mice and attenuated hypertrophy with induction of autophagy [39]. Data also indicated that miR-761 expression was reduced during Ang II-induced proliferation of VSMCs, and exogenous miR-761 delivery effectively inhibited the Ang II-induced VSMC proliferation. [288]. Experimental evidence showed that miR-761 directly targets mTOR and reduced its abundance [288]. Similarly, miR-99a was shown to negatively regulate hypertrophy through mTOR signaling pathway [185]. Interestingly, mice displayed an increase in mTOR activity starting at first week through 8 weeks following TAC- (transverse aortic constriction-) induced cardiac hypertrophy [185]. Overexpression of miR-99a suppressed mTOR and attenuated cardiac hypertrophy and cell death in TAC mouse model. Overexpression of miR-99a attenuated cardiac hypertrophy in TAC mice and cellular hypertrophy in cardiomyocytes subjected to Ang II or isoprenaline (ISO) through suppression of expression of mTOR [185].

In contrast, it has been shown that cardiac-specific overexpression of miR-222 induced pathological cardiac remodeling and heart failure in mice [289]. Transgenic mice with cardiac-specific expression of miR-222 (Tg-miR-222 mice) developed severe cardiac fibrosis and apoptosis, which led pathological cardiac remodeling and heart failure. The autophagy was inhibited in the hearts of Tg-miR-222 mice with activation of mTOR, but the expression of p27 was downregulated in the hearts of Tg-miR-222 mice [289]. It was suggested that miR-222 induced autophagy through activation of both mTORC1 and C2 complexes as shown with a substantial increase in both p-mTOR and p-S6 (Ser240/244) in transgenic Tg-miR-222 mice [289]. In the context of these findings, Su et al. also reported a role for p27-mTOR in the development of cardiac hypertrophy [290]. Cardiac-specific overexpression of miR-221, driven by the *α*-myosin heavy chain, resulted in hypertrophic hearts at 4 weeks of age with increased expression levels of ANP and BNP [290]. Moreover, miR-221 also inhibited autophagy, as demonstrated by downregulation of LC3-1/LC3-II ratio and an increase in p62 expression level [290]. Further, miR-221 overexpression in H9C2 cells and in primary cardiomyocytes showed decreased autophagosome formation as demonstrated with low number of EGFP-LC3 puncta [290]. More importantly, phosphorylation levels of mTOR and its substrates phospho-mTOR (S2448), phospho-4EBP1 (T37/46), and phospho-S6 (S235/236) levels were all significantly increased in Tg-miR-221 hearts at 4 weeks of age compared with those in the nontransgenic controls [290]. Conversely, silencing miR-221 in H9C2 cells and cardiomyocytes decreased the levels of phospho-mTOR, phospho-S6K, and phospho-S6, thereby establishing a link between miR-221 and mTOR signaling in the induction of cardiac hypertrophy [290]. Similarly, miR-365 was shown to promote cardiac hypertrophy through inhibition of autophagy by suppressing S-phase kinase-associated protein 2 (SPK2), an important activator of autophagy [291]. Conceptually, it was demonstrated that Spk2 induces autophagy through inhibition of mTORC1 and reverses adverse effect of cardiac hypertrophy [291]. Notably, Ang II treatment of cardiomyocytes increased the phosphorylation of the mTORC1 downstream effectors S6K and 4EBP1 and decreased the level of Spk2. Inhibition of mTOR activation, using rapamycin, completely abolished the Ang II-mediated inhibition of autophagy via miR-365-Spk2-dependent mechanism [291].

Recent studies have suggested that a long noncoding RNA (lncRNA), myocardial infarction-associated transcript (MIAT), plays a role in vascular remodeling and cardiac hypertrophy [41]. In this study, the authors demonstrated a three way link between MIAT, miR-93, and mTOR network. The upregulation of MIAT was associated with the decrease in miR-93 in Ang II-induced cardiac hypertrophy in rat [41]. Furthermore, it was shown that MIAT positively regulated TLR4 expression by acting as a sponge for miR-93 expression [41]. Overexpression of miR-93 attenuated MIAT-induced increase of TLR4 level in cardiomyocytes and attenuated Ang II-induced cardiac hypertrophy. In contrast, MIAT knockdown or miR-93 overexpression led to a significant inhibition on the protein levels of PI3K, p-AKT, and p-mTOR and blunted Ang II-mediated cardiac hypertrophy [41]. This study also suggested a strong corelation between miR-93, TLR4, and mTOR signaling, since overexpression of TLR4 enhanced the expression of miR-93 and blocked the protection observed with p-mTOR inhibition [41].

High fat diet (HFD) consumption for a prolonged time induces cardiac hypertrophy [292], and mTOR being a nutrition sensor plays an active role in mediating this effect in the heart [293]. Microarray analyses of the heart tissue of mice on HFD for 8 and 20 weeks identified a role for miR-451 in the development of cardiac hypertrophy [103]. Calcium-binding protein 39 (Cab39) is a direct target of miR-451 and an upstream kinase of AMP-activated protein kinase (AMPK). Suppression of miR-451 protected neonatal rat cardiac myocytes against palmitate-induced lipotoxicity through a mechanism that involves Cab39 [103]. In addition, cardiomyocyte-specific miR-451 knockout mice were resistant to HFD-induced cardiac hypertrophy. Protein levels of Cab39 and phosphorylated AMPK were increased, and phosphorylated mTOR and S6 phosphorylation were significantly suppressed in cardiomyocyte of the HFD-fed miR-451 cKO mice compared with control mouse hearts [103]. These findings elucidated an interesting aspect of AMPK-miR-451 and mTOR cross talk in cardiac hypertrophy.

Angiogenesis is an important process that plays a detrimental role in post-MI, and its abnormal regulation leads to cardiac hypertrophy [294]. mTOR and its downstream target AKT have been involved in the control of angiogenesis process during I/R injury [295–298]. Placental growth factor (P1GF), a member of vascular endothelial growth factor (VEGF) family, has been shown to induce cardiac angiogenesis and leads to hypertrophic heart [299]. Cardiac-specific overexpression of P1GF induced cardiac angiogenesis with increased expression of miR-182 at 6 weeks onset of angiogenesis process [299]. The study also found blunting of miR-182 upregulation in PlGF-induced eNOS^−/−^ mice, suggesting that miR-182 acts through NO-independent pathway to regulate angiogenesis [299]. Since NO exerts its function through AKT, it was further shown that mTORC1 was involved in the induction of angiogenesis and cardiac hypertrophy. Suppression of miR-182 using antimiR-182 decreased the phosphorylation of AKT^Ser473^ and p70-S6K^Thr389^, thus indicating an important regulatory effect of miR-182 on the AKT/mTORC1 pathway [299].

Endothelial cell dysfunction contributes to coronary vascular tone and results in atherosclerosis by affecting various growth factors, such as vascular endothelial growth factor, fibroblast growth factors (FGFs), and platelet-derived growth factors [300, 301]. PI3K/AKT/mTOR pathway plays a role in endothelial function and in the development of atherosclerosis [302]. miR-126 has been shown to play a role in alleviating oxidized low-density lipoprotein (ox-LDL) induced HUVEC injury through suppression of AKT-mTOR pathway [303]. The overexpression of miR-126 reversed ox-LDL-induced cell injury and apoptosis in HUVECs [303]. Conceptually, treatment of HUVECs with ox-LDL increased the phosphorylation of mTOR through activation of PI3K and AKT, and miR-126 mimics restored the impaired autophagic flux via inhibition of PI3K/AKT/mTOR pathway [303].

A recent study by Bera et al. revealed a significant role of miR-214 in the activation of mTORC1 that contributed to high-glucose-induced mesangial and proximal tubular cell hypertrophy and fibronectin expression [268]. miR-214 expression is increased in the renal cortex of type 1 diabetic mice. High glucose treatment induced the expression of miR-214 and decreased its target, PTEN, in mesangial and proximal tubular epithelial cells [268]. Suppression of PTEN subsequently increased the AKT-dependent mTORC1 activation to induce mesangial and proximal tubular cell hypertrophy and fibronectin expression. Quenching of miR-214 expression inhibited high-glucose-stimulated cell hypertrophy and expression of the matrix protein fibronectin. In contrast, overexpression of miR-214 suppressed PTEN and increased AKT activity similar to high glucose and led to phosphorylation of two mTORC1 inhibitors, PRAS40 and tuberin, which contributes to high-glucose-stimulated mTORC1 activation [268].

Interestingly, a recent study demonstrated that overexpression of lncRNA Plscr4 alleviated pressure overload-induced cardiac hypertrophy in mice and attenuated the increased cell surface area of cultured neonatal mouse cardiomyocytes treated with Ang II [304]. The study identified that Plscr4 elicits the antihypertrophic effects by repressing the prohypertrophy gene miR-214. Mitofusin 2 (Mfn2), which is located at the mitochondrial outer membrane, plays a negative regulator of cardiac hypertrophy by modulating mitochondrial fusion [305, 306]. Mfn2 is a direct target of miR-214 in the hypertrophic heart [307]. The interaction between Plscr4 and miR-214 attenuated the inhibitory effects of miR-214 on Mfn2. The overexpression of Plscr4 rescued the decreased expression of Mfn2 by sponging miR-214 in response to hypertrophic stress and, therefore, resisted mitochondrial dysfunction to alleviate hypertrophic growth [307]. However, the exclusive interplay between lncRNA Plascr4 and mTOR in regulation mediated by miR-214 of cardiac hypertrophy is yet to be identified.

## 3. Therapeutic Potential of miRNA and mTOR Inhibitors in CVD

Rapamycin, received the FDA approval in 1999, has been successfully used as an effective immunosuppressant post-organ transplantation to prevent allograft rejection [308]. The rapamycin-eluting coronary stent received first FDA approval in 2003 for use in coronary-artery stents to prevent restenosis [309–311]. Rapamycin is also used clinically for some rare forms of cancer (pediatric and adult patients with subependymal giant cell astrocytoma (SEGA), progressive neuroendocrine tumors of pancreatic origin (PNET), and SEGA associated with tuberous sclerosis (TS)) (http://www.cancer.gov/cancertopics/druginfo/fda-everolimus) [312, 313]. Multiple clinical trials of rapamycin are currently underway for several other disease conditions including lymphangioleiomyomatosis (LAM) [314], other metabolism modulating interventions on the elderly (NCT02874924), ALS (amyotrophic lateral sclerosis) (NCT03359538), Sturge-Weber syndrome (SWS) (NCT03047980), and type 1 diabetes (NCT01060605; NCT00014911-both are completed) [315, 316].

Rapalogs, the modified form of rapamycin, are widely considered in clinical trials for its anticancer property. In fact, the National Cancer Institute has registered more than 200 clinical trials involving either rapamycin or modified form of rapamycin both as monotherapy and as combination treatment cancer (NCT01698918; NCT00337376; NCT00930930) [317–320]. Due to the successful outcome of rapamycin in the clinical trials, several drugs analogs of rapamycin with modified chemical structure such as sirolimus, temsirolimus (CCI-779), everolimus (RAD001), and ridaforolimus (AP-23573) are being evaluated for enhanced treatment of several diseases [321–323]. In 2009, everolimus received approval from the FDA for HER2-negative breast cancer (advanced HR+ BC) patients in combination with exemestane after failure of a nonsteroidal aromatase inhibitor (Afinitor: Highlights of Prescribing Information). (http://www.accessdata.fda.gov/drugsatfda_docs/label/2012/022334s016lbl.pdf).

Current mTOR inhibitors available in the market are not complex-specific and can either partially suppress mTORC1 or completely block mTORC1 as well as C2. Therefore, several pharmaceutical companies ventured to develop second generation of mTOR inhibitor that can block both mTORC1 and C2. These inhibitors are designed to completely block the core catalytic activity of mTOR by acting as an ATP-competitive agents to mTOR subunit. On the contrary, diseases like cancer and cell cycle irregularities need specific inhibition of mTORC1 without interfering the activity of mTORC2. Since mTORC1 is vital for basic cellular process, it is indispensable, and its complete inhibition may lead to unwanted side effects. To overcome these obstacles, scientists are also in pursuit of developing inhibitor that target rictor or raptor to silence either mTORC1 or C2. Although mTOR inhibitors are promising drug for cancer treatment and immunosuppressant, an unmet clinical trial is essential for their therapeutic use in cardiovascular diseases. Substantially, evidences from laboratory models and preclinical trials suggest that mTOR inhibition in the heart is beneficial and prevents cell apoptosis [146, 158] and autophagy [324–327]. Interestingly, inhibition of mTOR by rapamycin or other rapalogs are shown to alter the expression pattern of miRs in the cardiovascular system [162, 199, 208, 228]. Especially, alteration of miRs through mTOR inhibition that changes the expression level of PTEN and other downstream targets can offer new treatment strategies [328]. Many miRNA-based therapies for cancer are in clinical trial and have shown efficiency in reducing tumor malignancy [312]. Mimic of miR-34 are currently being tested in phase I clinical trials (NCT01829971) for its anticancer properties [329]. It was demonstrated that low level of miR-34 is an indicator of poor prognosis in osteosarcoma (OS) patients. Sirolimus increases the sensitivity of human OS cells to anticancer drugs *in vitro* by upregulating miR-34b and suppressing its target p21-activated protein kinase 1 (PAK1) and ATP-binding cassette subfamily B member 1 (ABCB1) [329]. In contrast, the miR-34 family (miR-34a, -34b, and -34c) is upregulated in the heart in response to stress, including myocardial infarction or pressure overload via TAC [330]. Diabetes also increases the expression of miR-34a both in the heart and in circulation [331]. miRNA Therapeutics Inc. is developing an LNA-modified antimiR against miR-34a, which attenuates MI-induced remodeling and dysfunction, and also improves cardiac function and increase angiogenesis with activation of AKT in a model of pressure overload-induced pathological hypertrophy and dysfunction [330]. Mechanistically, miR-34 can directly target protein phosphatase PH domain leucine-rich repeat protein phosphatase (PHLPP2), a negative regulator of the PI3K/AKT/mTOR pathway.

Upregulation of miR-92a was shown to activate PI3K/AKT/mTOR pathway and inhibit cell apoptosis induced by chemotherapy in mantle cell lymphoma (MCL) cells [332]. Downregulation of miR-92a could inhibit the growth of tumors in a xenograft MCL mouse model [332]. Interestingly, pharmaceutical company Miragen developed MRG-110, an inhibitor of miR-92a, to enhance the revascularization process in ischemic heart disease. However, inhibition of angiogenesis is the goal for cancer therapy, and it should be assumed that miR-92a acts differently in cardiovascular system [333].

MGN-1374, a miR-15 inhibitor, is under the developmental stage by miRagen Therapeutics for treating myocardial infarction [334, 335]. Studies conducted in MDA-MB-231 breast cancer cells demonstrated overexpression of miR-15b/16 led to inhibition of cell proliferation causing G1 cell cycle arrest as well as caspase-3-dependent apoptosis by directly suppressing mRNA levels of RPS6KB1 and mTOR [336]. In addition, miR-15 was shown to regulate CD4^+^ regulatory T cells (Tregs) expression, which is essential for preventing autoimmunity. Overexpression of miR-15b/16 significantly enhanced the induction of Tregs in *Dicer*^−/−^ CD4^+^ T cells and suppressed the mTOR expression as evident with the decrease in phosphorylation of its downstream target, ribosomal protein S6 [337].

Cardiac expression of miR-208 was upregulated upon Ang II treatment and induced obesity through upregulation of mTORC1 in Zucker obese (ZO) rats [338]. Whereas, rapamycin treatment attenuated weight gain despite leptin resistance by attenuating the expression of miR-208 and increasing the expression of cardiac mediator complex subunit 13 (MED13), a suppresser of obesity, in ZO rats [338]. In addition, therapeutic inhibition of miR-208 prevents pathological cardiac remodeling, which coincides with a significant improvement in survival and cardiac function during heart disease [339]. MED13 is negatively regulated by a heart-specific miR-208a [340]. In this context, MGN-9103 (a LNA-modified antisense oligonucleotide against a cardiac-specific miR-208LNA) is a novel potential therapeutic candidate developed by miRagen Therapeutics for the treatment of obesity, diabetes, and metabolic syndrome and to improve cardiac function and survival rates during heart failure (http://drugprofiles.informa.com/drug_profiles/18925-mgn-9103). These research findings and clinical trials described above highlight the potential of miRNA-based therapies with an emphasis on mTOR signaling. Although several studies established a clear synergistic effect of miRNA and mTOR in the treatment of cancer, there are scarce reports of clinical trial in cardiovascular field. Nevertheless, conceptual treatments in laboratory models describing mTOR inhibition mediated miR changes and vice versa are encouraging and may lead to novel treatments in cardiovascular diseases in the future.

## 4. Conclusion

The role of mTOR in controlling the cellular dynamics in cardiovascular system provides confidence to consider mTOR and its related kinases as targets for therapeutic intervention. Most remarkably, changes in epigenetic signature of miRs upon mTOR inhibition can lead to identify novel miRNA-based treatment for cardiovascular diseases. Moreover, antagomir-based treatment options can specifically target individual mTOR complex and eliminate common side effects seen with dual mTOR inhibitors. Further understanding of the interfunctional relationship between mTORC1 and C2 complexes and its association with miRNA is warranted to develop an efficient miRNA-based therapeutics and diagnostics in cardiovascular system.
